# Major Depressive Disorder and Lifestyle: Correlated Genetic Effects in Extended Twin Pedigrees

**DOI:** 10.3390/genes12101509

**Published:** 2021-09-26

**Authors:** Floris Huider, Yuri Milaneschi, Matthijs D. van der Zee, Eco J. C. de Geus, Quinta Helmer, Brenda W. J. H. Penninx, Dorret I. Boomsma

**Affiliations:** 1 Department of Biological Psychology, Vrije Universiteit, 1081 BT Amsterdam, The Netherlands; m.d.vander.zee@vu.nl (M.D.v.d.Z.); eco.de.geus@vu.nl (E.J.C.d.G.); q.helmer@vu.nl (Q.H.); di.boomsma@vu.nl (D.I.B.); 2 Amsterdam Public Health (APH) Research Institute, Amsterdam, The Netherlands; y.milaneschi@ggzingeest.nl (Y.M.); b.penninx@amsterdamumc.nl (B.W.J.H.P.); 3 Department of Psychiatry, Amsterdam UMC, Vrije Universiteit, 1081 HJ Amsterdam, The Netherlands

**Keywords:** major depressive disorder, lifestyle, extended twin pedigree, variance decomposition, Mendel, genetic correlation, pleiotropy

## Abstract

In recent years, evidence has accumulated with regard to the ubiquity of pleiotropy across the genome, and shared genetic etiology is thought to play a large role in the widespread comorbidity among psychiatric disorders and risk factors. Recent methods investigate pleiotropy by estimating genetic correlation from genome-wide association summary statistics. More comprehensive estimates can be derived from the known relatedness between genetic relatives. Analysis of extended twin pedigree data allows for the estimation of genetic correlation for additive and non-additive genetic effects, as well as a shared household effect. Here we conduct a series of bivariate genetic analyses in extended twin pedigree data on lifetime major depressive disorder (MDD) and three indicators of lifestyle, namely smoking behavior, physical inactivity, and obesity, decomposing phenotypic variance and covariance into genetic and environmental components. We analyze lifetime MDD and lifestyle data in a large multigenerational dataset of 19,496 individuals by variance component analysis in the ‘Mendel’ software. We find genetic correlations for MDD and smoking behavior (*r*_G_ = 0.249), physical inactivity (*r*_G_ = 0.161), body-mass index (*r*_G_ = 0.081), and obesity (*r*_G_ = 0.155), which were primarily driven by additive genetic effects. These outcomes provide evidence in favor of a shared genetic etiology between MDD and the lifestyle factors.

## 1. Introduction

It is widely observed that multiple complex human traits tend to co-occur at the population-level. Klein and Riso (1993) presented a series of models explaining the causes of such comorbidity, which were extended by Neale and Kendler (1995) [1,2,3]. Explanations include chance and sampling bias, overlapping diagnostic criteria, multiformity where one disorder is an epiphenomenon of the other disorder and the co-morbid condition being an independent disorder. Complex traits can also have partly similar etiological processes, either environmental or genetic. The latter is defined as genetic pleiotropy [4], where one or multiple genes affect multiple traits, so that if the gene is segregating it causes simultaneous variation in the traits it affects. In recent years, evidence has accumulated with regard to the ubiquity of pleiotropy across the genome [5,6,7], and shared genetic etiology is thought to play a large role in the widespread comorbidity among psychiatric disorders [8,9]. Understanding genetic pleiotropy benefits our understanding of disease etiology, elucidating the relations among disorders as a function of sharing common genetic variant risk, as well as clarifying which traits and disorders are more distinct from one another [10].

In behavior genetics and genetic epidemiology, pleiotropy can be investigated by estimating the genetic correlation between traits. Similar to how the heritability of a trait encompasses the relative proportion of phenotypic variation in a population at a given time that is due to variation at the genetic level [11], the genetic correlation reflects the degree to which two traits share genetic variance [9]. The genetic correlation can be estimated through methods that analyze the co-segregation of traits in large extended pedigrees, such as those that are available in animal or plant breeding studies [4], or in human twin and family studies [12,13]. These methods employ the knowledge on genomic sharing from biometrical theory and base analyses on the cross-relative cross-trait covariance structure in bivariate phenotype data.

Major depressive disorder (MDD) is a complex, prevalent and burdensome condition with a well-established link with multiple lifestyle factors [14,15], including smoking behavior [16], physical (in)activity [17], and obesity [18]. Epidemiological data show smoking rate to be increased in clinically depressed individuals at twice the rate of the general population [19,20], and both population-based studies as well as prospective cohort studies find elevated risk of MDD in those who smoke [21,22,23]. A similar pattern has been found for physical inactivity, with increased risk for depression in the physically inactive [24,25,26], and reduced rates of physical activity in clinically depressed individuals [27,28,29]. Finally, both cross-sectional [30,31] and longitudinal studies [32,33,34] find evidence for bidirectional effects between depression and obesity.

The models proposed by Klein and Riso (1993) provide a range of explanations for these associations. In this paper we focus on exploring the shared genetic and environmental etiology of MDD and lifestyle traits, applying a bivariate biometrical approach to a large extended twin pedigree dataset of lifetime MDD and smoking behavior, physical inactivity, and obesity. We aimed to decompose phenotypic variance of these traits and their covariance with MDD into genetic and environmental components, thereby quantifying the degree to which comorbidity is explained by genetic and non-genetic contributions.

## 2. Materials and Methods

### 2.1. Participants and Procedures

We collected data on Major Depressive Disorder (MDD) and lifestyle variables in multi-generation twin families that are registered with the Netherlands Twin Register (NTR). Over the past three decades, the NTR recruited twins and their families for the study of human health and behavior. Methods of data collection include survey studies, experimental studies and biological sampling, as described in detail elsewhere [35]. Every two to three years, adult participants are approached with surveys pertaining to demographic information, phenotype data, and familial relations. The twelfth survey was collected in the period of 2015–2020 as part of the ongoing BIObanks Netherlands Internet Collective (BIONIC), a large consortium of which the NTR is a partner. BIONIC developed an online instrument to diagnose MDD in the Netherlands (detailed in [36]). In total, 21,823 surveys were collected by the NTR (1.2% paper-based, 98.9% online), with valid lifetime MDD data for 21,243 individuals. Informed consent was obtained before proceeding to the questionnaire items. During a pilot phase in 2015 and shortly after, the survey was distributed as a paper-based version.

The survey was collected in twins and their relatives. Opposite-sex twins are always dizygotic. Zygosity in same-sex twins was determined from genotype data (55.5%), or information from self-, parent-, or co-twin report. With this information, zygosity could be determined with certainty for 66.3% of twins. For the remaining twin pairs, zygosity was estimated from items on physical similarity, which we showed to capture DNA-confirmed zygosity 93% of the time [35]. Information on nuclear family and pedigree structure was obtained across multiple NTR databases (see [37]). We define a nuclear family as the combination of two parents and their offspring. As offspring proceed to become parents themselves, one individual can become part of multiple nuclear families. A pedigree refers to a collection of familial relations among individuals of, for example, a single nuclear family, or multiple nuclear families within large multi-generational pedigrees. The relations within a pedigree are not exclusively biological, as is the case for, e.g., spouses or adopted offspring.

### 2.2. Phenotypic Measures

BIONIC developed the Lifetime Depression Assessment Survey (LIDAS), an online self-report instrument for lifetime MDD ascertainment [36]. LIDAS is based on the Composite International Diagnostic Interview short form (CIDI-sf; [38]), and designed to efficiently identify lifetime MDD in population-based cohorts, in accordance with DSM-5 criteria. Bot et al. (2017) estimated sensitivity and specificity of LIDAS to be 85 and 80% respectively [36].

Lifetime major depressive disorder (MDD) status was determined in accordance with DSM-5 criteria from LIDAS data on nine symptoms [39]. Diagnostic criteria included having at least one of two core symptoms and having at least five of nine accessory symptoms, where symptoms were continuously present for a period of at least two weeks and caused significant disruption in daily functioning. Individuals without lifetime MDD (controls) were defined as having fewer than five symptoms, no core symptoms, and no significant disruption in daily functioning. Controls were further screened for the presence of other psychiatric disorders. If no diagnosis or treatment for a psychiatric disorder were reported, these individuals were included as ‘screened controls’. Other controls (*n* = 1747) were excluded, together with individuals with unknown sex (*n* = 7), age < 16 (*n* = 3), and insufficient symptom data for diagnosis (*n* = 570).

Smoking behavior was recorded with three answers (0 = non-smoker, 1 = current smoker, 2 = former smoker) and was dichotomized (0 = ‘never smoked’, 1 = ‘ever smoked’). Physical inactivity was recorded as the number of times per week respondents engaged in physical activities in their leisure time that caused sweating, with responses ranging from 0 (‘None; zero times’) to 1 (‘Yes, once per week’), 2 (‘Yes, twice per week’), 3 (‘Yes, three times per week’), and 4 (‘Yes, four times per week or more’). Answers were recoded into three levels of physical inactivity, so that 0 = ‘three or four times per week or more’, 1 = ‘once or twice per week’, and 2 = ‘zero times per week’. Body-mass index (BMI) was defined as weight in kg divided by length in meters squared. Extreme BMI values were excluded if weight < 45 kg or > 200 kg, height < 150 cm or > 220 cm, or BMI < 15 or > 50. BMI data of 157 participants were retrieved from earlier surveys. For obesity, continuous BMI data were binned into categories so that 0 = unaffected (BMI < 30) and 1 = obese (BMI > 30), in accordance with WHO criteria.

### 2.3. Genetic Analyses

To obtain indications of familial resemblance in lifetime MDD the lifestyle traits, we computed within- and cross-trait familial correlations for multiple pairs of relatives [40]. These included MZ (monozygotic) and DZ (dizygotic) twin pairs, siblings, spouses and parent-offspring, for all possible female-male combinations (e.g., brothers, sisters, father-son, father-daughter, mother-son, mother-daughter), taking into account that an individual may contribute to multiple correlations (e.g., one mother may have two daughters in the dataset, creating two mother-daughter pairs).

Univariate and bivariate variance component analyses were conducted in the ‘Mendel’ software v. 16.0 [41]. Mendel is a versatile toolset for the statistical analysis of complex traits and incorporates an enhanced version of the variance components program ‘Fisher’ [42] for classical biometrical analyses. Mendel obtains maximum likelihood estimates of parameters and submodels can be compared through likelihood ratio tests [43]. Pedigree data with diverse types of relatives allows for the decomposition of phenotypic (co)variance into its underlying genetic and environmental components [44,45,46], especially when data of monozygotic twin pairs are included. Table 1 summarizes the genetic relatedness of relative pairs included in the pedigree, with their expected proportion of genetic sharing based on biometric theory [47].

When size and complexity of pedigrees increase, specification of genetic relations among relatives can become increasingly difficult. The Mendel software allows inclusion of complex family (co)relations in large irregular pedigrees, at a cost of freedom in model specification. The specification of familial relations is achieved in a single input pedigree file, where one row corresponds to one individual, and where the first six columns contain all the required information to specify genetic relations: Family ID, Person ID, Father ID, Mother ID, Sex, and Twincode (to accommodate MZ twin pairs). All members of an (extended) pedigree share a family ID, while each individual in the pedigree has a unique personal identifier. Mendel requires either both parents or neither of them to be specified, and dummy parents were created to complete nuclear families where one parent was missing [37]. An additional field allows specification of which family members share a household. Sharing of a home environment may create resemblances over and above the resemblance that is explained by genetic relatedness. The definition of a household is flexible, in that sharing can be specified for an entire nuclear family or limited to specific relative pairs (e.g., siblings or spouses). Only one household variable can be specified, which then applies to all phenotypes in a model, so that careful consideration is required which definition is most appropriate for the phenotype, or phenotypes, in a bivariate model. We specified household sharing for all members of a nuclear family—spouses, their offspring, and the sibling relations between offspring—if offspring were under 24 years of age, 24 being the average age at which offspring leave their parental home in the Netherlands [48].

The Mendel software treats all phenotypes as quantitative. Categorical variables were scaled in such a way that higher categories reflected a higher score. In the genetic analyses, we consider obesity both as a dichotomous measure (BMI < 30 vs. BMI ≥ 30) and a continuous variable (BMI). BMI was log transformed to eliminate skewness. In all analyses, sex, age, and age^2^ were added to the linear regression model as fixed effects. Age^2^ was included to account for non-linear effects of age on MDD. Age was standardized before its quadratic term was computed, effectively reducing their correlation to approximately zero [49].

**Figure 1:**
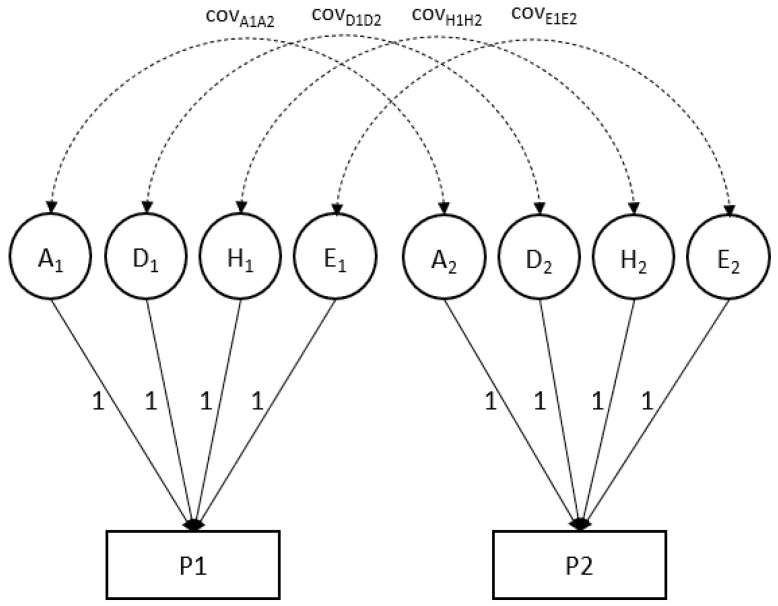
Illustration of a bivariate model, where the variance of each phenotype (P1 and P2) is due to four factors (A, D, H, E), and the covariance (cov) reflects the variance that is shared between the A, D, H and E factors. We estimated the variance of the latent factors and the covariances indicated in the figure.

The residual phenotypic (co)variances were decomposed into four variance components: additive genetic variance (A), non-additive genetic variance (D), household variance (H), and unique environmental variance (E). The bivariate model is illustrated in Figure 1. Variance components and their standard errors were estimated by maximum likelihood and estimates of genetic and environmental correlation were obtained from the raw maximum likelihood covariance and variance component estimates (Box 1).

Box 1 **The Bivariate Model.**

  The bivariate model represented for an individual for two phenotypes (P1 and P2):  P1 = G1 + NG1 = A1+ D1 + H1 + E1  P2 = G2 + NG2 = A2+ D2 + H2 + E2  Var (P1) = Var(G1) + Var (NG1)  Var (P2) = Var(G2) + Var (NG2)  Broad-sense heritability (P1) = Var(G1)/Var (P1) = *H*^2^  Narrow-sense heritability (P1) = Var(A1)/Var (P1) = *h*^2^  Covar (P1, P2) = Covar (G1, G2) + Covar (NG1, NG2)  Phenotypic correlation: *r* (P1, P2) = Covar (P1, P2)/*SD*(P1) × *SD*(P2)  Genetic correlation: *r* (G1, G2) = Covar (G1, G2)/*SD*(G1) × *SD*(G2);where P is an individual’s phenotypic value (possibly a
residual after correction for fixed effects of, e.g., age and sex), G is
genotypic value and NG stands for non-genetic value. Var(P) is the variance
of the phenotype (or the phenotypic residual); var(G) and var(NG) stand for
genetic and non-genetic variance components (assuming no covariance of G and
NG). G can be decomposed into additive genetic (A) and non-additive
(dominance; D) values; non-genetic influences can be distinguished into those
that are common to members from the same household (called household effects
(H) in Mendel) and all other (unique; E) environmental effects.  The covariance between two phenotypes, here labeled P1
and P2 (e.g., MDD and smoking) likewise can be decomposed into genetic and
non-genetic covariance. The correlation of P1 and P2 is obtained by scaling
the phenotypic covariance by the product of the standard deviations of P1 and
P2. Likewise, the genetic correlation is obtained by dividing the genetic
covariance by the standard deviations of G1 and G2.

## 3. Results

### 3.1. Phenotypic Overview

The sample throughout the analyses consisted of 19,496 individuals (4300 lifetime MDD cases, 15,196 healthy controls), for which a descriptive overview is provided in Table 2. The sample consisted of 12,535 females (64.3%) and 6961 males (35.7%), with ages ranging from 16 to 92 and a mean of 41.75 (*SD* = 16.61). Females were on average 40.60 (*SD* = 15.98) years old, and males were on average 43.81 (*SD* = 15.98) years old. There were 10,799 nuclear families with at least one individual with phenotype data. The sample contained 9261 twins and 10,235 non-twin individuals. The 9261 twins (47.2% MZ, 52.8% DZ, 0.3% unknown zygosity) comprised 2375 complete twin pairs and 4482 unpaired twin individuals.

**Figure 2:**
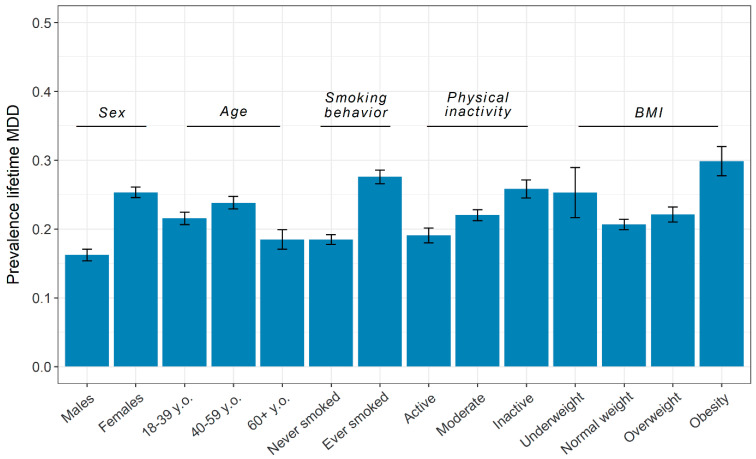
Prevalence of lifetime major depressive disorder across levels of demographic and lifestyle factors. MDD = major depressive disorder; BMI = body-mass index. Error bars reflect a 95% confidence interval.

Figure 2 displays prevalence of lifetime MDD across demographic and lifestyle categories. As expected, lifetime MDD is higher in women than in men, and is more prevalent in middle-aged than in younger persons, but as we reported earlier [50], it is lower in the 60+ age group. Figure 2 also shows a more unfavorable profile for all lifestyle traits in lifetime MDD cases than in controls. Affected persons smoke more often, they have a higher body weight and they are more often physically inactive. Correlations among the lifestyle variables were positive and weak: smoking behavior and physical inactivity (*r* = 0.046), smoking behavior and BMI (*r* = 0.163), physical inactivity and BMI (*r* = 0.126).

### 3.2. Kinship Correlations

We estimated within- and cross-trait kinship correlations for various relative pairings, listed in Table 3. If genetics contribute to familial similarity, we expect phenotypic kinship correlations to decrease with decreasing genetic similarity among more distant kinship pairs. We observed a consistent pattern where relatives who were genetically more similar showed higher within-trait correlations across all considered traits. For example, the within-trait correlation of lifetime MDD equaled *r* = 0.439 and *r* = 0.299 in MZ males and females and ranged between *r* = 0.072 and *r* = 0.303 in DZ and sibling pairs, suggesting that a genetic component contributed to familial resemblance of MDD. Table 3 also contains the spousal correlations for two groups, i.e., parents of twins and twins with their own spouses. Parents of twins were older (median age = 44 years) than twins and their spouses (median age = 27 years). We computed spousal correlations separately for these groups. There was little evidence for differences in resemblance between the younger and older spouse groups. For MDD, spousal correlations were small (*r* = 0.105 vs. *r* = 0.050). The largest difference between the two age groups for BMI (*r* = 0.272 in twins and their spouses vs. *r* = 0.185 in parents of twins). Cross-trait correlations between MDD and smoking showed a pattern where MZ twin correlations were larger than those in first-degree relatives, suggestive of a genetic contribution to their comorbidity. Such patterns were less evident for the other trait combinations.

### 3.3. Variance Component Analyses

We first conducted a series of univariate variance component analyses, decomposing the phenotypic variances of lifetime MDD, smoking behavior, physical inactivity, BMI and obesity into four variance components: additive genetic variance (A), non-additive genetic variance (D), household variance (H), and unique environmental variance (E). Maximum likelihood estimates of variance components and their standard errors are listed in Table 4. All variance component estimates of lifetime MDD and lifestyle variables were significantly larger than zero, indicating contributions of genetic and non-genetic factors to all traits. Estimates of broad-sense heritability (*H*^2^), that is, the sum of the additive and non-additive genetic variance components divided by the total phenotypic variance, were *H*^2^ = 0.335 in MDD, *H*^2^ = 0.550 in smoking behavior, *H*^2^ = 0.318 in physical inactivity, *H*^2^ = 0.725 in BMI and *H*^2^ = 0.647 in obesity. Thus, these broad-sense estimates combine the influence of additive and non-additive genetic effects. The magnitude of non-additive genetic effects (dominance; D) varied across traits. The magnitude of household effects (H) was consistently low but significant across traits, suggesting that shared household effects play a minor role in the phenotypic resemblance among relatives who live together. A large proportion of phenotypic variance was due to unique environment i.e., individual-specific environmental factors (E) and measurement error, particularly in lifetime MDD (E = 0.618) and physical inactivity (E = 0.574).

Next, bivariate analyses were conducted for MDD and each of the lifestyle factors. Covariance component and correlation estimates are listed in Table 5. We found positive genetic correlations (*r*_G_) between lifetime MDD and smoking behavior (*r*_G_ = 0.249), physical inactivity (*r*_G_ = 0.161), BMI (*r*_G_ = 0.081), and obesity (*r*_G_ = 0.155). The positive directions of these genetic correlations indicate that the genetic factors that are shared between traits tend to influence both trait values in the same direction; the shared genetic etiology contributes either to an increase or a decrease in both traits, but not an increase in one and a decrease in the other.

Unique to an extended twin pedigree design, we were able to delineate genetic correlations into additive and non-additive genetic components. Estimates of additive genetic correlations (*r*_A_) tended to be larger than non-additive genetic correlations (*r*_D_), and most non-additive genetic covariances included zero in their 95% confidence interval (10.96 times the standard error). Additive and non-additive genetic components were typically correlated in the same (positive) direction. The genetic correlation with lifetime MDD was stronger for obesity (*r*_G_ = 0.155) than for BMI (*r*_G_ = 0.081), suggesting that the genetic correlation of body weight and lifetime MDD may be stronger when differentiating between normal and more extreme cases of body weight.

Household components for lifetime MDD and lifestyle were not correlated, as household covariances did not reach significance for any of the trait combinations. A correlation between household components would suggest that some aspects of sharing a household can cause similar changes in two traits. Some estimates of *r*_H_, such as those between MDD and smoking behavior (*r*_H_ = 0.158), and MDD and BMI (*r*_H_ = 0.146), were larger than others. However, the main effects of household, defined as the contribution of non-genetic factors that increase familial resemblance, were very modest to begin with. Finally, unique environmental effects explained considerable trait variance, but correlations between unique environmental components (*r*_E_) were close to zero, ranging from *r*_E_ = 0.005 in BMI to *r*_E_ = –0.043 in obesity. This suggests that although unique environmental effects can have a large effect on individual differences in both lifetime MDD and lifestyle variables, few of these effects are shared across the traits considered here. Estimates of E incorporate non-systematic effects and measurement error, which likely are uncorrelated, and so the low estimates of *r*_E_ are not unexpected.

## 4. Discussion

We sought to quantify the genetic and non-genetic contributions to comorbidity between lifetime Major Depressive Disorder (MDD) and three indicators of lifestyle: smoking behavior, physical inactivity, and obesity. We conducted bivariate variance component analyses in data of twins and extended family relations from the Netherlands Twin Register, decomposing phenotypic variance and covariance into additive genetic (A), non-additive genetic (D), household (H), and unique environmental (E) components. Covariance estimates between trait components were scaled by their respective variance estimates to obtain estimates of genetic and environmental correlations. We found genetic correlations between MDD and all lifestyle factors. In contrast, there was little evidence for correlations between household or unique environmental effects. That is, phenotypic relations between MDD and the lifestyle traits were primarily driven by genetic effects, with considerable additive genetic correlations (*r*_A_) for all MDD-lifestyle combinations.

We found a broad-sense heritability for MDD of 33.5%, in line with previous estimates from twin data (34–37%) [51,52], and register-based data with reconstructed extended familial relationships (25–32%) [53,54]. The most current SNP-*h*^2^ estimate for MDD, defined as the percentage of phenotypic variation that is due to variation in common single nucleotide polymorphisms (SNPs), equals 8.9% [55,56]. Environmental effects on MDD were primarily individual-specific (E; 62%), as is consistent with the literature. In contrast to many other studies, we observed that common environment, here defined as household sharing (H), played a minor but significant role in explaining individual differences in MDD, with around 5% of variance accounted for by these effects. Also of interest is that few twin and family studies of MDD report estimates of non-additive effects (D), whereas we find roughly equal additive and non-additive genetic contributions to the broad-sense heritability (making up 18.9% and 14.6% of total phenotypic variance, respectively). Similar increases in non-additive genetic effects have been reported in extended twin pedigree designs for other traits (e.g., [40,57,58]), and may result from increases in statistical power or the ability to model additional variance components. We note that the inclusion of non-twin family relations introduces age differences within relative pairs, which might mimic non-additive effects when, for example, age differences result in reduced resemblance in parent-offspring pairs, but not in DZ twin or sibling pairs (Table 1). However, a longitudinal analysis of depression data found no evidence for genetic innovation, i.e., after adolescence there was no evidence that different genes were expressed at later ages, and so we do not expect to see artificial non-additivity [59].

Turning to the bivariate analyses of MDD and lifestyle factors, we may also compare our results of modest genetic correlations with findings from twin and SNP-based studies. Findings from twin studies vary with regard to the genetic correlation between MDD and smoking behavior. Some studies report moderate to large genetic correlations in the range of *r*_G_ = 0.25–0.56 [21,60,61], whereas others find no evidence for a genetic correlation [62,63], or instead find evidence for a shared environmental etiology [64]. Similar to SNP-*h*^2^, genetic correlation estimates can be derived from molecular data (SNP-*r*_G_) [9]. The most recent SNP-*r*_G_ estimate between MDD and ‘ever vs. never smoked’ equals SNP-*r*_G_ = 0.314 (95% CI: 0.242–0.385) [55]. Our estimate (*r*_G_ = 0.249) lies at the low end of this confidence interval. For physical inactivity, findings from an earlier bivariate twin study suggest a moderate genetic correlation between depressive symptoms and exercise behavior (*r*_G_ = –0.230) [65], and a more recent study finds a negative SNP-*r*_G_ between MDD and physical activity of SNP-*r*_G_ = –0.100 [29]. We find a genetic correlation in the expected opposite direction, as we use physical inactivity as the outcome, that is somewhat in between these two estimates (*r*_G_ = 0.161). Finally, twin studies provide some evidence for a shared genetic etiology between MDD and BMI or obesity. Afari et al. (2010) applied a bivariate twin method to depression and obesity data and found a genetic correlation of *r*_G_ = 0.120 [66]. However, Choy et al. report a significant proportion of shared environmental factors between depression and BMI, but no significant genetic correlation [67]. The most recent SNP-*r*_G_ estimate for depression and BMI equals SNP-*r*_G_ = 0.076, and those between depression and obesity equal SNP-*r*_G_ = 0.086, 0.082, and 0.168 for obesity class 1, 2, and 3, respectively [55]. Our estimates of genetic correlation with MDD are similar to these SNP-*r*_G_ estimates, with *r*_G_ = 0.081 for BMI and *r*_G_ = 0.155 for obesity.

These results should be viewed in light of some limitations. First, the extended twin pedigree design relies on a number of assumptions which, when violated, could bias parameter estimates [58]. However, the method is a statistically powerful approach [68] that relies on fewer assumptions than, e.g., the classical twin design, and has been suggested to be more robust to violations of these assumptions [69]. The possibility to analyze all family relations from large extended pedigrees in the Mendel software came at a cost of restrictions in model specification, so that some effects (e.g., sex-dependent heritability, gene-environment interaction or correlation) could not be modeled. Likewise, only a single definition of household effects could be specified for two traits in a bivariate model. Ideally, we would have specified a unique and best-suiting definition of household separately for each trait and possibly for different sets of relatives. We also note that household is a form of the common environment that may not take into account any lasting effects of having shared a household. This may have contributed to the low estimates of household effects in univariate models, although modest estimates for shared environmental effects are consistent with the literature. Further, we recognize that the lifestyle factors we consider are not independent from each other, although correlations between them were weak.

Interpreting these findings requires a consideration of the mechanisms that can underlie phenotypic relations at the population level, and the mechanisms that can underlie correlations at the genetic level. Well-established associations exist for MDD and smoking [16], physical inactivity [25,70], and BMI and obesity [18,32], but findings regarding the underlying mechanisms tend to support different functional mechanisms. These mechanisms include causal effects from one trait to another, bidirectional causality between two traits, or confounding by a third set of factors, such as a shared genetic or environmental etiology (e.g., [1,60]). Likewise, a genetic correlation between phenotypes can still indicate multiple mechanisms that include causality and pleiotropy [71,72]. One distinction is that of horizontal and vertical pleiotropy. In horizontal pleiotropy, a gene affects multiple phenotypes directly and independently from each other. This occurs when, for example, a gene product is a precursor of multiple physiological end-products. Indeed, such a mechanism is congruent with the mechanism of genetic confounding mentioned above. In contrast, vertical pleiotropy arises when a genetic variant affects one trait that in turn affects a second trait in a cascade-like manner, i.e., indirect causality between the gene and the second trait. In this case, two traits share a genetic etiology only because there exists a causal relation between the two. Both pleiotropic mechanisms lead to genetic correlation, but have different implications for our understanding of etiological mechanisms, risk assessment, disease prediction, and treatment and prevention strategies [73,74,75]. An important direction of future research is to distinguish between such mechanisms.

Here, we find genetic correlations, but environmental correlations of nearly zero. Together, these findings suggest that insomuch as the associations between lifetime MDD and lifestyle are explained by causal effects, they also reflect a partially shared genetic etiology. Distinguishing between etiological and pleiotropic mechanisms, which need not be mutually exclusive, is no easy task, and caution should be applied in inferring causality or the absence thereof. However, we do note that under causality, we would have expected both genetic and environmental effects to translate from one trait to the other [65].

## 5. Conclusions

In summary, we find that phenotypic relations in the Dutch population between lifetime MDD and smoking behavior, physical inactivity, and obesity are partly driven by a shared genetic etiology. We demonstrate how estimates of genetic and environmental correlation can be derived in an extended twin pedigree design, with analyses conducted in the Mendel software. We show how pedigree analyses can serve as an alternative and feasible means to studying the shared etiology of disease and potential risk factors, and how genetic correlation estimates from extended twin pedigree data triangulate with similar estimates from molecular genetic data to benchmark pleiotropic effects.

## Figures and Tables

**Table 1:** Familial relative pairs and expected proportion of genetic sharing.

Pairing	Additive Genetic Sharing	Dominance Sharing
Monozygotic twins	1	1
Dizygotic twins and non-twin siblings	0.5	0.25
Three-quarter siblings	0.375	0
Half-siblings	0.25	0
Parent-offspring	0.5	0
Grandparent-grandchild	0.25	0
Aunt/uncle-niece/nephew	0.25	0
First cousins	0.125	0
First cousins with monozygotic twins as parents	0.25	0
Double first cousins	0.25	0.0625
Spouses	0	0

**Table 2:** Demographic and phenotypic descriptives of extended twin pedigree data. MDD = major depressive disorder.

		Age, Years	Sex		MDD	
	*N*	Mean (*SD*)	Female	Male	Screened Control	Case
Total	19,496	41.75 (16.61)	12,535 (64.3%)	6961 (35.7%)	15,196	4300
MDD, *N* (%)						
Screened control	15,196 (77.9%)	41.72 (16.93)	9363 (74.7%)	5833 (83.8%)	-	-
Case	4300 (22.1%)	41.82 (15.43)	3172 (25.3%)	1128 (16.2%)	-	-
Smoking behavior, *N* (%)						
Never smoked	11,760 (60.4%)	37.76 (15.98)	7860 (62.8%)	3900 (56.1%)	9588 (63.2%)	2172 (50.5%)
Ever smoked	7715 (39.6%)	47.80 (15.70)	4661 (37.2%)	3054 (43.9%)	5588 (36.8%)	2127 (49.5%)
Physical inactivity, *N* (%)						
Active	5052 (25.9%)	37.96 (17.06)	2901 (23.2%)	2151 (30.9%)	4089 (26.9%)	963 (22.4%)
Moderate	10,220 (52.4%)	42.53 (16.10)	6760 (53.9%)	3460 (49.7%)	7971 (52.5%)	2249 (52.3%)
Inactive	4214 (21.6%)	44.36 (16.52)	2869 (22.9%)	1345 (19.3%)	3126 (20.6%)	1088 (25.3%)
Body-mass index *						
Underweight (<18.5)	550 (2.8%)	25.99 (12.46)	393 (3.2%)	157 (2.3%)	411 (2.7%)	139 (3.3%)
Normal weight (18.5–24.9)	11,557 (59.8%)	38.28 (16.51)	7722 (62.2%)	3855 (55.4%)	9169 (60.8%)	2388 (56.0%)
Overweight (25–29.9)	5459 (28.2%)	48.56 (14.55)	3053 (24.6%)	2406 (34.7%)	4252 (28.2%)	1207 (28.3%)
Obesity (≥30)	1768 (9.1%)	48.97 (13.28)	1240 (10.0%)	528 (7.6%)	1240 (8.2%)	528 (12.4%)

* This categorization is for illustrative purposes; the analyses are of continuous BMI and obesity.

**Table 3:** Within- and cross-trait kinship correlation estimates for various kinships with valid lifetime major depressive disorder data in the extended twin pedigree. MDD = major depressive disorder; PI = physical inactivity; BMI = body-mass index; MZ = monozygotic twins; DZ = dizygotic twins.

	*r* (*n*)					*r* between MDD Relative 1; Lifestyle Relative 2				*r* between Lifestyle Relative 1; MDD Relative 2			
Kinship	MDD	Smoking	PI	BMI	Obesity	Smoking	PI	BMI	Obesity	Smoking	PI	BMI	Obesity
Spouse (parents)	0.050 (1570)	0.259 (1563)	0.167 (1568)	0.185 (1556)	0.096 (1556)	0.009	0.023	–0.004	0.001	–0.004	0.021	–0.018	–0.020
Spouse (twins)	0.105 (386)	0.227 (384)	0.155 (384)	0.272 (384)	0.061 (384)	0.047	0.017	0.014	0.065	–0.048	0.025	–0.107	–0.094
MZ males	0.439 (342)	0.505 (341)	0.411 (342)	0.726 (339)	0.548 (339)	0.085	0.008	0.003	0.034	0.164	0.031	–0.051	0.001
MZ females	0.299 (986)	0.561 (983)	0.376 (984)	0.764 (979)	0.490 (979)	0.132	0.043	0.019	0.036	0.132	0.051	0.045	0.054
DZ males	0.303 (195)	0.260 (194)	0.212 (194)	0.224 (193)	–0.027 (193)	–0.006	0.097	–0.022	–0.094	0.152	0.048	–0.053	0.130
DZ females	0.072 (410)	0.246 (410)	0.194 (409)	0.293 (400)	0.045 (400)	0.064	0.047	0.034	0.027	0.046	0.112	0.005	0.002
DZ opposite sex	0.139 (450)	0.223 (449)	0.104 (449)	0.299 (443)	0.139 (443)	0.057	–0.016	0.060	0.112	0.119	–0.012	0.022	0.013
Mother-Daughter	0.105 (2440)	0.128 (2437)	0.135 (2440)	0.296 (2409)	0.161 (2409)	–0.005	0.029	0.049	0.051	0.052	0.024	0.030	0.023
Mother-Son	0.092 (1324)	0.173 (1322)	0.039 (1324)	0.205 (1314)	0.092 (1314)	0.042	–0.007	–0.040	–0.027	0.035	–0.014	0.002	0.003
Father-Daughter	0.111 (1700)	0.113 (1699)	0.148 (1699)	0.229 (1680)	0.134 (1680)	–0.012	0.017	0.031	0.053	0.022	0.065	0.010	–0.011
Father-Son	0.103 (1007)	0.201 (1007)	0.110 (1007)	0.253 (1001)	0.086 (1001)	0.077	–0.004	0.0003	–0.027	–0.010	0.027	–0.004	0.017
Brother-Brother	0.235 (202)	0.307 (201)	0.238 (201)	0.315 (197)	0.112 (197)	–0.041	–0.026	0.025	–0.025	0.158	0.130	0.016	0.157
Brother-Sister	0.143 (1128)	0.176 (1127)	0.080 (1126)	0.256 (1120)	0.121 (1120)	–0.010	0.020	0.066	0.077	0.089	0.031	0.015	–0.031
Sister-Sister	0.091 (579)	0.296 (578)	0.206 (578)	0.334 (564)	0.169 (564)	0.131	0.058	0.015	–0.019	0.072	0.088	0.041	–0.028

**Table 4:** Maximum-likelihood (co)variance component estimates and derived correlation estimates between major depressive disorder and four lifestyle factors: smoking behavior, physical inactivity, body-mass index, and obesity. All models included sex, age, and age^2^ as fixed covariates. MDD = major depressive disorder; PI = physical inactivity; BMI = body-mass index; VC = variance component; se = standard error; A = additive genetic component; D = non-additive genetic component; H = household component; E = unique environment component.

Model	*N*	Raw VC (se)				Standardized VC			
		A	D	H	E	A	D	H	E
MDD	19,496	0.032 (0.005)	0.025 (0.006)	0.008 (0.003)	0.105 (0.004)	0.189	0.146	0.048	0.618
Smoking	19,475	0.033 (0.007)	0.088 (0.007)	0.028 (0.003)	0.071 (0.003)	0.149	0.401	0.127	0.323
PI	19,486	0.038 (0.014)	0.109 (0.016)	0.049 (0.007)	0.264 (0.010)	0.082	0.236	0.108	0.574
BMI	19,334	0.009 (0.001)	0.006 (0.001)	0.002 (0.0003)	0.004 (0.0002)	0.422	0.303	0.095	0.175
Obesity	19,334	0.015 (0.003)	0.0384 (0.003)	0.009 (0.001)	0.021 (0.001)	0.185	0.462	0.107	0.246

**Table 5:** Maximum-likelihood (co)variance component estimates and derived correlation estimates between major depressive disorder and four lifestyle factors: smoking behavior, physical inactivity, body-mass index, and obesity. All models included sex, age, and age^2^ as fixed covariates. MDD = major depressive disorder; PI = physical inactivity; BMI = body-mass index; VC = variance component; se = standard error; A = additive genetic component; D = non-additive genetic component; H = household component; E = unique environment component; G = genetic component, where A + D = G.

Model	*N*	Raw Covariance (se)				Correlation				
		A	D	H	E	A	D	G	H	E
Smoking and MDD	19,475	0.008 (0.004)	0.013 (0.005)	0.002 (0.002)	–0.001 (0.003)	0.238	0.278	0.249	0.158	–0.006
PI and MDD	19,486	0.012 (0.006)	0.002 (0.007)	0.001 (0.003)	–0.004 (0.005)	0.357	0.044	0.161	0.061	–0.022
BMI and MDD	19,334	0.003 (0.001)	–0.0003 (0.0013)	0.001 (0.001)	0.0001 (0.0007)	0.160	–0.024	0.081	0.146	0.005
Obesity and MDD	19,334	0.006 (0.003)	0.003 (0.003)	–0.0002 (0.001)	–0.002 (0.002)	0.256	0.093	0.155	–0.023	–0.043

## Data Availability

Data are available from the Netherlands Twin Register upon reasonable request (https://tweelingenregister.vu.nl/information_for_researchers/working-with-ntr-data, accessed on 20 September 2021).
